# Interaction between common variants of *FTO* and *MC4R* is associated with risk of PCOS

**DOI:** 10.1186/s12958-015-0050-z

**Published:** 2015-06-02

**Authors:** Huiqin Yuan, Guoping Zhu, Fang Wang, Xiang Wang, Huihui Guo, Mo Shen

**Affiliations:** Department of Gynaecology and Obstetrics, First Affiliated Hospital, Huzhou Teachers College, the First People’s Hospital of Huzhou, Huzhou, 313000 China; Department of Laboratory Medicine, First Affiliated Hospital, Huzhou Teachers College, the First People’s Hospital of Huzhou, Huzhou, 313000 China; Department of Laboratory Medicine, First Affiliated Hospital of Wenzhou Medical University, Wenzhou, 325000 China

**Keywords:** *FTO*, *MC4R*, Genetic association study, Polycystic ovary syndrome

## Abstract

**Background:**

Polycystic ovary syndrome (PCOS) is a common and complex endocrine-metabolic disease. One of the well-documented characteristics of PCOS is obesity or overweightness. It is possible to be genetically predisposed to becoming obese or overweight, and several potentially causative single nucleotide polymorphisms (SNPs), such as rs9939609 (A/T) in the fat mass, and obesity-associated gene (*FTO*) and rs17782313 (T/C) in the melanocortin-4 receptor gene (*MC4R*), have been investigated. Further investigation of association between obesity-associated SNPs and PCOS susceptibility will contribute to a better understanding of the disease.

**Methods:**

In the present study, we enrolled 733 patients with PCOS and 892 control subjects. The common variants *FTO* rs9939609 and *MC4R* rs17782313 were genotyped and their relationship with obesity-related traits was evaluated.

**Results:**

Rs9939609 and rs17782313 are associated with PCOS and obesity-related traits and profiles. The association found between PCOS and *FTO* rs9939609 (*p* = 0.0302) was attenuated after adjustment for BMI (*p* = 0.187). *MC4R* rs17782313 did not confer an increased risk for PCOS (*p* = 0.368) even after adjustments (*p* = 0.715). Interestingly, the interaction of *FTO* and *MC4R* polymorphisms was more significantly associated with PCOS (*p* = 0.031, adjusted for age and BMI). The *FTO* variant rs9939609 is associated with Chinese women with PCOS; however, this association is affected by BMI.

**Conclusions:**

The combined pathogenic effect of *FTO* and *MC4R* polymorphisms indicates a direct role of the interaction between *FTO* and *MC4R* polymorphisms in the development of PCOS.

## Background

Polycystic ovary syndrome (PCOS), which affects 6 to 8 % women of reproductive age, is diagnosed by clinical and/or biochemical androgen excess, menstrual irregularity and polycystic ovaries. Additionally, PCOS patients are characterized by an increased risk of insulin resistance, type 2 diabetes mellitus, and overweightness/obesity [1–4]. Fifty percent of PCOS patients are overweight or obese [5], and increasing evidence supports obesity as being an important factor in the etiology of PCOS [6, 7]. The development of obesity is affected by both genetic factors and environmental aspects, which is similar to the development of PCOS [8–10]. It is possible that a shared genetic predisposition contributes to their co-occurrence. Scrutinizing obesity-associated genes in PCOS patients may help to explain the etiology of both obesity and PCOS, and identification of susceptibility genes for either condition has increased with the aim of eliminating the effects of obesity in PCOS.

The fat mass and obesity-associated gene (*FTO*), located on chromosome *16q12.2*, has been identified by large-scale genome-wide association studies and is recognized to be associated with both body mass index (BMI) and obesity [11, 12]. The human *FTO* gene is expressed in adipose tissue, muscle, the pancreas, the liver and adipose tissue, with the highest concentrations found in the hypothalamus [13]. It has been reported that the *FTO* gene is associated with eating habits and is important in the control of energy homeostasis [14]. rs17782313, located 188 kb downstream of *MC4R*, was similarly associated with obesity [15, 16]. Additionally, the *MC4R* gene is the most common genetic cause of human obesity in inherited morbid obesity. *In vitro* studies have shown that *MC4R* is a key regulator in central melanocortin neuronal pathways [17]. Association studies have revealed the connection between PCOS and SNPs in *FTO* and *MC4R*, respectively. Regarding *FTO*, rs9939609, rs1421085, rs17817449, rs8050136 have been found to be associated with PCOS, among which rs9939609 is the most extensively studied and is the only successfully replicated SNP in Han Chinese populations [18–25]. In a recent genome-wide association study conducted in Han Chinese women, rs9939609 marginally achieved genome-wide significance (2.47E-03) [26]. rs17782313 and rs9939609 in *MC4R* were also studied in patients with PCOS [18, 19]. However, understanding obesity and overweightness, as well as PCOS, require consideration of both gene-gene interactions and case-control differences in BMI. Recently, the combined effect of *FTO* rs9939609 and *MC4R* rs17782313 has been discussed in relation to obesity, breast cancer and endometrial cancer [16, 27, 28]. The above-mentioned studies revealed that an association exists between the interaction of *FTO* rs9939609 and *MC4R* rs17782313 and these independent diseases, which implied a combined effect of these two SNPs. The role of rs9939609 of *FTO* and he rs17782313 of *MC4R* in women with PCOS is of interest. In the present study, we chose two representative SNPs, *FTO* rs9939609 and *MC4R* rs17782313, to investigate whether there is a combined effect of the two SNPs in the development of PCOS in a Chinese Han population.

## Methods

### Ethics statement

The study was approved by the Institutional Review Board of the First Affiliated Hospital of Wenzhou Medical University (IRB reference number: 2011–21, initiating and ending dates: 06/2011–06/2014). Written informed consent was obtained from all participants.

### Subjects

A total of 733 PCOS patients were recruited from the First People’s Hospital of Huzhou from Jun 2011 to May 2014. Diagnosis of PCOS was based on the Rotterdam Consensus proposed in 2003 [29], which consists of meeting at least two of the following criteria: oligo-/ovulation, polycystic ovarian morphology, and clinical or biochemical hyperandrogenism (hyperandrogenism, HA). The diagnosis of PCOS was made only when the other etiologies for hyperandrogenemia and ovulatory dysfunction were excluded, i.e.*,* congenital adrenal hyperplasia, 21-hydroxylase deficiency, androgen-secreting tumors, Cushing’s syndrome, thyroid disease, and hyperprolactinemia. The total testosterone and mF-G scores were evaluated for hyperandrogenism.

A total of 892 women who were referred for routine physical examination or tubal factor infertility were enrolled during the same time period. All subjects in the control group had a regular menstrual cycle (26–35 days) and normal ovarian morphology. Additionally, their total testosterone and mF-G score were evaluated. Written informed consent was obtained from all participants.

All participants resided in Zhejiang province, were unrelated and at reproductive age with no hormonal therapy for at least 3 months. The medication and weight loss histories of all subjects were collected to exclude those who were taking hypoglycemic agents or hormonal therapy.

### Clinical and biochemical measurements

Height and weight were measured while participants were dressed in underwear without shoes. BMI was calculated as the weight in kilograms divided by the square of the height in meters. Obesity was defined as BMI > =28 kg/m^2^, and overweight was BMI > =24 kg/m^2^, according to Chinese criteria [30]. The waist circumference was measured midway between the lowest rib and the iliac crest. The hip circumference was the longest measurement around the hips. The waist-hip ratio (WHR) was defined as the waist circumference divided by the hip circumference.

Peripheral blood samples were tested on day 2–4 of spontaneous cycles or after cessation of bleeding from all the subjects after a 12 h overnight fast. The follicle stimulating hormone, luteinizing hormone, prolactin, testosterone and estradiol levels of all subjects were measured with a chemiluminescent analyzer (Beckman Access Health Company, Chaska, MN, USA).

### SNP genotyping

Peripheral blood samples were obtained from all participants and collected into tubes treated with the anti-coagulant EDTA. Genomic DNA was extracted using a QIAamp DNA mini kit (QIAGEN, Hilden, Germany) according to the manufacturer’s protocol. Polymerase chain reaction (PCR) was performed with the primers for *FTO* rs9939609 (forward: 5′-AAGAGATGATCTCAAATCTACTTTATGAGATA-3′; reverse: 5′-TTAGAGTAACAGAGACTATCCAAGTGCATCAT-3′; annealing temperature 56 °C) and primers of *MC4R* rs17782313 (forward: 5′-AGGAAACAGCAGGGATAGGG-3′; reverse: 5′-TGCTGAGACAGGTTCAT AAAAAG-30, annealing temperature 56 °C). The PCR products were purified and sequenced on an ABI 3700 automated sequencer (Applied Biosystems, Foster City, CA, USA). Five percent of randomly selected samples were re-sequenced to validate the genotyping assays.

### Statistical analysis

Continuous parameters of patients and controls are expressed as means ± SD. PLINK (v.1.07, http://pngu.mgh.harvard.edu/purcell/plink) was applied to calculate the Hardy-Weinberg equilibrium (HWE), allele frequency differences and genotype differences. Because there were no significant deviations of the allele frequencies from HWE (*p* > =0.05), genotype and allele frequencies were compared. A power calculation was performed using a Genetic Power Calculator [31]. Genetic models were divided into additive (+/+ vs. +/- vs. -/-), dominant (+/+ plus +/- vs. -/-) and recessive (+/+ vs. +/- plus -/-). Logistic regression for the disease trait was conducted to exclude the potential confounding effects of age and body mass index. The genotype models were applied for the phenotype analysis, which was compared by one-way ANOVA or student *T* test using SPSS software (SPSS version 16, IL, USA). *p* < 0.05 was considered statistically significant.

## Results

### Clinical characteristics of subjects

Seven hundred and thirty-three PCOS patients and 892 controls were recruited, and the case-control genetic power reached 0.8 at the *p* = 0.001 level. Clinical measurements are presented in Table 1. In the PCOS group, 20.6 % (150/733) of subjects were obese (BMI > =28 kg/m^2^), while the percentage in the control group was only 10.1 % (90/892). Additionally, 44.1 % (320/733) of PCOS women were overweight or obese (BMI > =24 kg/m^2^), while the percentage in the control group was 21.9 % (196/892). There were no differences in the waist circumference and WHR after age and BMI were adjusted by logistic regression.

**Table 1:** Comparison of basic clinical characteristics between PCOS and control subjects

Characteristics	PCOS	Control	*P*	*P adj*
AGE	26.14+/−3.23	29.38+/−4.53	<0.001	-
BMI	25.16+/−5.27	22.73+/−2.97	<0.001	-
WAIST	84.75+/−10.19	78.04+/−7.38	<0.001	0.21
WHR	0.87+/−0.06	0.84+/−0.04	<0.001	0.79
FSH	6.15+/−1.71	7.47+/−2.95	<0.001	<0.001
LH	9.82+/−5.63	4.89+/−2.29	<0.001	<0.001
T	56.61+/−22.90	21.55+/−12.51	<0.001	<0.001

*SD* standard error, *WHR* waist-hip ratio, *FSH* follicle-stimulating hormone, *LH* luteinizing hormone, *T* testosterone, *P adj* adjusted *P* value by age and BMI in logistic regression

### Allele and genotype frequency

The observed genotype distributions of the two SNPs were consistent with Hardy-Weinberg equilibrium. The minor allele frequency (MAF) comparison is presented in Table 2. The P value of rs9939609 is 0.030 (*p* = 0.187; *OR* = 1.27; *95 % CI*: 1.02–1.58), which was attenuated after adjusting for BMI. For rs17782313, no association with PCOS was observed (*p* = 0.368; *OR* = 1.07; *95 % CI*: 0.91–1.27), and it was also attenuated after adjusting for BMI. The genotype comparisons are shown Table 3. Of the three genotypes of rs9939609, the recessive genotype model was the most powerful (*p* = 0.020). No statistical significance was found for rs17782313.

**Table 2:** Allele comparison of rs9939609 and rs17782313

	Risk allele	MAF PCOS	MAF control	*P*	OR 95 % CI	*P adj*
rs9939609	A	0.126	0.102	0.030	1.27	0.187
					1.02–1.58	
rs17782313	C	0.242	0.229	0.368	1.07	0.715
					0.91–1.27	

*MAF* minor allele frequency, *OR* odds risk, *95 % CI* confidence interval, *P adj*, adjusted *P* value by BMI in logistic regression

**Table 3:** Allele and genotype analysis of rs9939609 and rs17782313

SNP	Comparisons	PCOS	Control	*P*	OR (95 % CI)
		Minor/Major	Minor/Major		
rs9939609 A/T	ADD	16/153/564	7/168/717	0.030	-
	DOM	169/564	175/717	0.091	1.23 (0.97–1.56)
	REC	16/717	7/885	0.020	2.82 (1.15–6.89)
rs17782313 C/T	ADD	35/285/413	27/354/511	0.187	-
	DOM	320/413	381/511	0.70	1.04 (0.85–1.27)
	REC	35/698	27/865	0.067	1.61 (0.96–2.68)

*ADD* additive genotype (three genotypes) study, *DOM* dominant genotype (homozygotes of risk allele + heterozygotes vs. homozygotes of non-risk allele) study, *REC* recessive genotype study (homozygotes of risk allele vs. heterozygotes + homozygotes of non-risk allele)

To eliminate the influence of BMI in the association between rs9939609, the PCOS and control subjects were further divided into an obese/overweight group and a non-obese/overweight group to eliminate the influence of BMI (Table 4). The MAF of the PCOS groups was higher (*OR obese/overweight* =1.31; *OR non-obese/overweight* =1.30); however, there was no significant difference between the PCOS and controls (*P obese/overweight* =0.074 and *P non- obese/overweight* =0.247). Moreover, there were MAF differences in the PCOS subjects with different BMIs (*p* = 0.005, *p adjusted* = 0.74), which is similar to the control group (*p* = 0.031, *p adjusted* = 0.21). The differences in the rs9939609 MAF between the PCOS and control groups could be explained by the influence of the BMI.

**Table 4:** rs9939609 allele comparisons of PCOS and control divided into groups according to the presence or absence of obesity

	MAF		*P*	OR 95 % CI
	PCOS	Control		
Obese group	0.16	0.13	0.074	1.31 (0.95–1.67)
Non-obese group	0.12	0.09	0.247	1.30 (0.92–1.68)

*P adj*, adjusted *P* value by BMI in logistic regression. The comparison of the PCOS Obese group and Non-obese group was carried out (*p* = 0.005, *p adj* = 0.74), which was also true for the controls (*p* = 0.031, *p adj* = 0.21)

To investigate the combined effect, we also studied the participants who were simultaneously carrying the risk A and C alleles of *FTO* or *MC4R*. The subjects were divided into a group with both risk alleles (simultaneously carrying the risk A and C, PCOS vs. control = 131 vs. 121), a group carrying one risk allele (carrying the risk A or C, PCOS vs. control = 227 vs. 314), and a group with no risk alleles. Additionally, the cluster of risk alleles has been found in PCOS subjects (*P group of risk allele* = 0.017, *OR* = 1.38), which remained even after adjustments for BMI (*p* = 0.031).

### Clinical and metabolic measurements of different genotypes in PCOS

According to the genetic analysis, there was an association between rs9939609 and PCOS, but not between rs17782313 and PCOS. Furthermore, a genetic-phenotype association study was carried out only for rs9939609. Clinical data, particularly metabolic and lipid indices, of PCOS patients are presented in Table 5 by the additive model of rs9939609. The BMI and waist circumference increased along with the presence of the risk allele A. However, the WHR was not different between the three groups. Basic clinical indices, such as FSH, LH and T, were higher in the AA group with no significant differences. The fasting insulin level of OGTT was higher among patients with the AA genotype. As the homeostasis model assessment-estimated insulin resistance (HOMA-IR) was introduced, the increasing trend, along with the A risk allele, was also observed.

**Table 5:** Clinical and metabolic data of rs9939609 PCOS patients in the additive model

	Genotype AA	Genotype AT	Genotype TT
AGE	26.82+/−4.19	26.16+/−3.65	26.40+/−3.77
BMI ^a, b, c^	26.49+/−4.01	25.16+/−4.23	24.70+/−4.41
WAIST ^a, b, c^	89.74+/−12.36	85.20+/−11.98	83.19+/−10.93
WHR	0.87+/−0.04	0.86+/−0.07	0.86+/−0.07
FSH	6.27+/−1.64	6.31+/−1.61	6.27+/−1.69
LH	11.35+/−5.29	10.73+/−5.89	10.54+/−6.81
T	55.92+/−25.69	53.54+/−25.18	54.82+/−24.32
GLU-0′	5.57+/−0.73	5.33+/−0.66	5.41+/−0.82
GLU-120′	7.73+/−1.74	6.62+/−2.01	6.65+/−2.19
INS-0′ ^c^	15.71+/−12.11	12.55+/−9.39	12.30+/−8.24
INS-120′	78.64+/−88.49	68.18+/−65.61	67.59+/−60.59
HOMA-IR	3.97+/−3.81	3.01+/−3.22	2.98+/−2.29
CHOL	4.53+/−0.90	4.51+/−0.92	4.55+/−0.90
TG	1.30+/−0.91	1.30+/−0.88	1.33+/−0.92
HDL	1.30+/−0.50	1.33+/−0.35	1.32+/−0.55
LDL	2.67+/−0.97	2.68+/−0.92	2.68+/−0.91

The data are presented as the mean ± SD

*GLU* glucose, *INS* insulin, *HOMA-IR* homeostasis model for insulin resistance, *CHOL* cholesterol, *TG* triglycerides, *HDL* high density lipoprotein, *LDL* low density lipoprotein

^a^ the comparison of genotype AA vs. genotype AT is significantly different (*p* < 0.05)

^b^ the comparison of genotype AT vs. genotype TT is significantly different (*p* < 0.05)

^c^ the comparison of genotype TT vs. genotype AT is significantly different (*p* < 0.05)

## Discussion

The mechanism underlying the association of obesity with PCOS remains unclear. Obesity is associated with hyperandrogenism and menstrual disturbance of PCOS and worsening complications, such as T2DM and hypertension [7, 4, 32]. However, some studies have argued that obesity in PCOS reflects environmental factors to a great extent [33] and that results may be influenced by BMI and other adipose-related measurements. Additionally, it is reasonable to study the pleotropic effects of BMI-associated alleles in PCOS to explain the increased BMI levels and weight in PCOS patients. The present study was designed to investigate if the common candidate genes for obesity are also related to PCOS. Additionally, the interaction of *FTO* and *MC4R* polymorphisms is associated with PCOS.

In the present study, we again observed an association between *FTO* and PCOS, although it was attenuated after adjustment for BMI. In one large-scale study in a Han Chinese population, the A allele for the *FTO* gene was significantly more frequent among PCOS patients than it was in the control population (14.1 % vs. 10.2 %; A allele vs. T allele, OR = 1.44, *P* = 1.86E-09), which was in accordance with our study [25, 22]. However, we did not replicate the results that *FTO* gene is a candidate gene for PCOS independent of BMI effects [22], potentially due to our limited sample size. The other study conducted in a Han Chinese population reported similar results. The association between *FTO* and PCOS was mediated by BMI [25]. Similar to rs9939609 of *FTO*, the rs17782313 of *MC4R* has been reported to contribute to elevated BMI in PCOS, but it does not appear to play a major role in PCOS [18]. It has also been reported that there is no association between rs17782313 and PCOS [18]. However, when the two SNPs are considered together, a polygenetic effect is evident and the association is seemingly more significant.

The *FTO* rs9939609 SNP is related to body weight through its influence on energy intake and satiety [34]. However, the underlying association of *FTO* polymorphisms with PCOS risk remains unclear. One recent meta-analysis suggested that the effect of the *FTO* polymorphism rs9939609 may not be associated with the risk of PCOS in the overall population [35], and we also concluded that there is a direct association between the *FTO* variant and PCOS risk that is independent of BMI (adiposity) in East Asians [25, 22]. In Asian populations, the MAF of rs9939609 is not as high as in Caucasian populations (43–49 %); in our study, the MAF was 12.6 % [22]. The different genetic background of different ethnic populations may explain the discrepancy in the MAF. Although a direct association between the *FTO* variant and PCOS risk has been found [20, 22] and successfully replicated in this study, the effect of the *FTO* gene on PCOS is possibly related to its genetic interaction with other susceptibility genes, considering there were limited PCOS patients carrying the risk allele. The combination of susceptibility genes may contribute to the polygenic background of PCOS. In the present study, we observed a significant association between *FTO* and *MC4R* with PCOS in a Chinese population (*p* = 0.0014).

PCOS is considered to be complex, multifactorial or polygenic, meaning that the disease is likely associated with the effects of multiple genes in combination with lifestyles and environmental factors. Traditional approaches for studying the genetic basis of PCOS have limitations because they only target a single gene or cluster of tightly linked genes. Genes located on different chromosomes could together contribute to the genetic background of PCOS. As in the scenario in the current study, the association of *FTO* with PCOS was successfully replicated, but the association of the *MC4R* polymorphism was modest. However, based on their similar functions in control of food intake and energy consumption, *FTO* may contribute to the susceptibility of PCOS in combination with *MC4R*, and patients who are positive for both the *FTO* and the *MC4R* risk alleles are more likely to suffer from PCOS than those patients who are positive for either the *FTO* or the *MC4R* risk alleles.

## Conclusions

A significant association was observed between PCOS and *FTO* rs9939609, but the association was attenuated after adjustment for BMI. However, *MC4R* rs17782313 was not related to an increased risk for PCOS, and the interaction of *FTO* and *MC4R* polymorphisms exhibited a more significant association with PCOS (*P* = 0.031, adjusted for age and BMI), indicating a combined pathogenic effect of *FTO* and *MC4R* in the development of PCOS.

## Abbreviations

BMI: Body mass index
FTO: Fat mass and obesity-associated gene
HA: Hyperandrogenism
HWE: Hardy-Weinberg equilibrium
LD: Linkage disequilibrium
MAF: Minor allele frequency
MC4R: Melanocortin-4 receptor gene
PCOS: Polycystic ovary syndrome
SNP: Single nucleotide polymorphisms
